# Microbial strain collections and information: An annotated selection of World Wide Web sites relevant to the topics in microbial biotechnology

**DOI:** 10.1111/1751-7915.12135

**Published:** 2014-06-05

**Authors:** Lawrence P Wackett

**Affiliations:** Department of Biochemistry Molecular Biology and Biophysics BioTechnology Institute University of MinnesotaSt. Paul, MN, 55108, USA

## National Collection of Industrial Microorganisms

### http://www.ncl-india.org/files/NCIM/

This culture collection contains only non-pathogenic microorganisms contains information on strain names and special application of the particular microorganisms.

## Collection of industrial and environmentally-relevant microorganisms

### http://www.ncimb.com

This collection specializes in microorganisms valued for industrial applications, includes many patented microbes, and the company also provides oilfield and environmental services.

## NCIMB bacteria database

### http://www.cabri.org/CABRI/srs-doc/ncimb_bact.info.html

This database provides a large set of industrial microorganisms. Under specific entries, it contains information on the origins of, and literature on, the strains.

## DSMZ

### http://www.dsmz.de

The DSMZ is the German collection of microorganisms and cell cultures. It contains approximately 20,000 bacterial and 5,000 fungal strains.

## Microbial strain information

### http://www.straininfo.net

Microbial strain information is a tool with the goal of providing a comprehensive microbial knowledge base.

## Fungal biodiversity center

### http://www.cbs.knaw.nl/Collections/

This database focus on fungi and bacteria of the class known as actinomycetes.

## Strain info: Reducing microbial data entropy

### http://www.ncbi.nlm.nih.gov/books/NBK92731/

This web article describes the needs and designs of compiling microbial strain information.

## World federation for culture collections

### http://www.wfcc.info/index.php/about/sites/

This clearinghouse site contains links to many microbial strain collections around the world.

## Ribosomal database project

### http://rdp.cme.msu.edu

The RDP provides annotated bacterial, archael, and fungal rRNA sequences for use in taxonomy and in analyzing secondary structure.

## Global catalogue of microorganisms

### http://gcm.wfcc.info

This site provides information for global culture collections to share and coordinate information.

## ATCC: Bacteria

### http://www.atcc.org/Products/Cells%20and%20Microorganisms/Bacteria.aspx

The American Type Culture Collection specializes in microorganisms useful for research, medical and industrial applications.

## Richer metadata for microbial sequences

### http://www.standardsingenomics.org/index.php/sigen/article/view/sigs.4851102/1109

There is a push to provide fuller data on strains to accompany genome sequences to allow users to more fully benefit from the genome data.

## NCBI taxonomy browser

### http://www.ncbi.nlm.nih.gov/Taxonomy/Browser/wwwtax.cgi

The NCBI taxonmy browser provides information on all taxonomic units including bacteria and archae.

## Prokaryotic names with standing in nomenclature

### http://www.bacterio.net

This site specializes in taxonomic information for prokaryotic strains.

## GOLD: Genomes online

### http://genomesonline.org/cgi-bin/GOLD/index.cgi

This database contains a large amount of information on microbial genomes as well as metadata on those strains.

## Database of magnetotactic bacteria

### http://database.biomnsl.com

This database focuses specifically on bacteria that use the magnetic field of the earth to navigate.

## List of biocatalysis/biodegradation microorganisms

### http://umbbd.ethz.ch/servlets/pageservlet?ptype=allmicros

This page lists the strains contained with the University of Minnesota Biocatalysis/Biodegradation Database. There are links to information on the metabolic pathways contained within those strains.

## Microbes online

### http://www.microbesonline.org

Microbes online contains information on microbial genomes, gene expression, and fitness.

